# Work-From-Home in the New Normal: A Phenomenological Inquiry into Employees’ Mental Health

**DOI:** 10.3390/ijerph20010048

**Published:** 2022-12-21

**Authors:** Mumtaz Ali Memon, Saba Shaikh, Muhammad Zeeshan Mirza, Asfia Obaid, Nuttawuth Muenjohn, Hiram Ting

**Affiliations:** 1 NUST Business School, National University of Sciences and Technology, Islamabad 44000, Pakistan; 2 Department of Management Sciences, National University of Modern Languages Hyderabad Campus, Hyderabad 71000, Pakistan; 3 School of Management, College of Business and Law, RMIT University, Melbourne 3000, Australia; 4 Department of Tourism and Commerce, UCSI University, Kuching 93000, Malaysia

**Keywords:** work from home, system complexities, mental health, COVID-19, qualitative

## Abstract

The COVID-19 pandemic has forced employees to adapt and adjust to the new normal in an unprecedented way. While some employees have been able to move to work-from-home (WFH) relatively easily, many find it challenging. Notwithstanding the magnitude of change, little is known about the determinants of WFH employees’ mental health during COVID-19. This study therefore aims to explore (1) the salient factors that contribute to the mental health issues of WFH employees and (2) strategies to overcome WFH challenges. A qualitative approach using phenomenological inquiry was adopted. Forty-one employees who worked from home in Pakistan were sampled using the purposive and snowball sampling techniques. Data was collected via semi-structured interviews and analyzed using thematic analysis. Overall, employees believe that organizations offer inadequate support in both work-related and non-work-related matters. Five themes were elicited and coded as factors that contribute to mental health issues among WFH employees. Technical issues and system complexities, the absence of flexible working arrangements, distractions, a lack of communication, and inadequate social support were found to obstruct WFH and cause mental distress. Behavioral and cognitive coping strategies were also determined to tackle these mental issues. This study complements the human resource literature by exploring the factors that obstruct WFH and cause mental health issues in the context of the pandemic crisis. As mental well-being is more intricate than administrative arrangements, the study is useful for organizations to develop a feasible mechanism that facilitates the smooth execution of WFH for employees while ensuring their mental health is preserved. Using a phenomenological inquiry, the present study is one of the few to explore the factors that contribute to the mental health of WFH employees in the context of the pandemic crisis. Apart from its contribution to knowledge on human resource management and organizational behavior, it provides useful implications for managers, policymakers, and practitioners to manage WFH employees more effectively.

## 1. Introduction

An unknown virus—later named COVID-19—hit the city of Wuhan, China in December 2019. It spread exponentially and caught other countries by surprise before it was declared a worldwide pandemic by the World Health Organization (WHO) in March 2020 [1]. To curb its spread and flatten the curve, lockdowns, movement controls, and physical distancing measures were imposed [2]. Due to health concerns and subsequent stay-at-home mandates by the authorities, many companies lost their customers and revenue, leaving them with no choice but to reduce, suspend, or adjust their operations and strategies [3]. As the pandemic has persisted with no sign of a permanent solution, the resolve of companies, especially their human resource personnel, has been greatly challenged. Therefore, to keep business going and make adjustments to navigate their human capital, work-from-home (WFH) procedures have been adopted by most organizations [4].

WFH is known by different names, such as telecommuting, remote working, and teleworking. All these terms refer to arrangements in which employees do not work at conventional offices, warehouses, or stores. Rather, they are free to work at a place that is convenient to them(i.e., home) [5]. While WFH is beneficial, it is also challenging as its working protocols differ from conventional working formats. Moreover, WFH during COVID-19 exposes an organization’s rigidity and employees’ vulnerabilities. For some job positions, the transition to WFH is relatively easy while for others, performing regular work tasks from home can be daunting [6]. Thus, though the pandemic has forced companies to swiftly make adjustments, it has also compelled employees to endure changes with uncertainty. In fact, it is projected that every four employees out of five, which encompasses almost 81% of the global workforce (3.3 billion), are badly affected by the complete or partial closure of businesses [7].

Geographically, Pakistan lies between epicenters of COVID-19, such as China and Iran. The first case of COVID-19 in Pakistan was detected on 25 February 2020, while the first death was reported on 29 March that year [8]. The single case reported in late February 2020 grew to 20 cases within two weeks, following which, the trajectory of COVID-19 infections in Pakistan surged over the next months. As a developing country, Pakistan has grappled with dealing with COVID-19, strained by its limited resources and an unstable economy [9]. The government began with a partial/smart lockdown (particularly in outbreak areas) and the closure of both major public and private sector organizations. The lockdown and quarantine measures marked the beginning of a unique set of inevitable challenges for both employers and employees, foremost of which was stress and anxiety, along with other dilemmas for the general population. Undoubtedly, COVID-19 has put the psychological health of millions at stake by triggering anxiety and apprehension. This was confirmed by a World Health organization (WHO) report which found that a large fraction of the global population, specifically in middle- and low-income countries, were suffering from psychological, neural, and substance use disorders [10]. Many studies have also highlighted mental health concerns among the workforce during the pandemic [11,12]. Therefore, it is important to explore the factors that affect the mental health of WFH employees in order to preserve their mental well-being and social harmony during COVID-19 and beyond.

The key objectives of this research were to: (1) explore the factors affecting the mental health of employees working from home during COVID-19; and (2) explore strategies to overcome WFH challenges. By fulfilling these objectives, this research adds to the current literature in four ways. First, the growing body of WFH literature on the COVID-19 pandemic appears to focus more on corporeal health issues, such as respiratory hygiene, social distancing, and herd immunity, rather than the mental problems people have faced during this pandemic [13,14,15]. However, past research has advocated that epidemics have severe and viable psychological effects on the community [16]. Discussing only physical health while neglecting mental health issues thus fails to portray the true picture of individuals’ general health [17].

Second, many studies have emphasized the psychological health of medical staff who are exposed to the direct risk of virus transmission [18,19]. For example, Spoorthy et al. [16] suggested regular medical screening for healthcare workers to relieve the stress associated with COVID-19. Nonetheless, further investigation is needed into the influence of COVID-19 on the mental health of WFH employees. As WFH employees do not work in a conventional workplace and have lower virus transmission risk, they could have different psychological concerns.

Third, WHO has stressed the need for nations to initiate interventions to cure the expected psychological consequences of this pandemic [10]. Likewise, Xiang et al. [20] stated that concerns related to physiological health call for practical implications to ensure individual mental well-being. However, to date, the specific mental health factors that stem from these consequences are unclear. Without understanding the factors that contribute to mental health problems, it is hard to cope with them. Therefore, the findings of this investigation will help policymakers formulate better strategies to tackle the mental health problems of WFH workers.

Lastly, past studies in the literature have mainly focused on the quantitative method. Quantitative analysis usually lacks insights into the subjective experiences of respondents [21,22,23]. Qualitative analysis, on the other hand, seeks to gather evidence-based knowledge, unearth contextual facts, and develop increased phenomenon awareness [24]. In this regard, a deeper exploration of employee experiences provides better insights into the research phenomenon. Therefore, this study attempted to qualitatively examine employees’ experiences with WFH and its impact on their mental well-being amid the ongoing pandemic.

This paper is structured as follows. First, we present a theoretical description of the research context to highlight the main issues in the available literature. In the next section, we explain the methodology employed in this study. Then, data analysis results are reported, following which a comprehensive framework is proposed based on the findings. A thorough discussion of the key findings is subsequently presented before outlining their theoretical and practical implications. Lastly, future directions and closing remarks conclude the paper.

## 2. Literature Review

Due to the coronavirus (COVID-19), we are exploring unchartered seas; in doing so, we are realizing the importance of managing, living, and working together for social well-being and stability [15]. In the business environment, the virus has changed the conception of work in contemporary organizations. Businesses have faced the most uncertain situations and, consequently, have been forced to take decisions which they never have done before. Specifically, the pandemic has altered prevailing working geographies by enforcing WFH practices for employees [25] as an essential part of the new normal since the beginning of the COVID-19 disruption [26]

WFH (also known as telecommuting) is a working arrangement that allows individuals to shift work from their office to their home [27]. It permits operating from home without normal arrangements and guidelines provided by direct supervisors and other structural sources. Since WFH employees work with greater autonomy and isolation while engaging in relatively less interaction with co-workers, WFH blurs the dividing line between one’s personal and professional lives [2].

Previous literature on WFH has advocated its potential benefits, which include increases in flexibility, efficiency, policy conformity, morality, and transparency, as well as reductions in instability, turnover, absenteeism, and fixed costs [28]. WFH also helps individuals work with greater freedom, as they are separated from the constant monitoring of peers and supervisors. In the long term, such autonomy builds their self-control [29]. The flexibility of scheduling linked to WFH has further beneficial consequences for personal and family life, which boosts satisfaction in the long run [30]. WFH also helps employees maintain a healthier work-life balance, which in turn increases their job efficiency.

Given its advantages, many studies have identified the personal and environmental antecedents of WFH [27,31]. Haines III et al. [27] revealed supervisor and technical support as environmental antecedents and self-management and motivation as personal antecedents of WFH. Work overload, work-life conflict, and role ambiguity have also been found as stressors that lead to undesired outcomes when working at home [32]. Notably, a review of 46 meta-analyses and 12,883 employees conducted by Gajendran and Harrison [33] concluded that telecommuting does not have a general adverse impact on the quality of employment relationships. Rather, they found that telecommuting has few but mostly beneficial impacts on lateral outcomes, such as better performance, job satisfaction, and perceived control, as well as lower turnover, role stress, and work-family conflict. It was also observed that these beneficial effects seem to be partly mediated by autonomy.

### 2.1. WFH during COVID-19 and Mental Health

WFH during COVID-19 has blurred the line that divides work roles from personal roles [34]. Additionally, the closure of schools and daycare centers has inflated the unexpected responsibilities of working parents. Augmented visibility and intensified childcare demands have forced parents, especially those working from home, to make severe adjustments [35]. Studies have found that females have endured more severe distress than their male counterparts during COVID-19 [21,36]. Another issue is related to role transition. To remain focused, it is imperative to leave one role and uninterruptedly enter the other. To do so, teleworkers have suggested that they signal themselves by leaving their ‘home’ space and entering a particular place allocated for ‘work’ [37].

During epidemics, the most critical concern of public health officials and the government typically pertains to the biological and physical impacts of the crisis, with far less attention given to mental health concerns [19]. However, psychological reactions to recent major outbreaks, in particular, the 2014 to 2016 Ebola virus (EVD) epidemic, provide an insight into the likely mental health impacts of quickly spreading diseases [38]. Concerns about mental illness, as expressed by Xiang et al. [20], call for widespread, realistic, and rigorous steps to maintain mental well-being in such times. Today, the current situation of COVID-19 has brought a loss of typical routine, a struggle to find a peaceful working environment, detachment from peers and friends, and an overload of work, which engender feelings of professional and social isolation [12,39,40]. Following such unprecedented disturbances, it is important to consider not only the visible consequences of COVID-19 but also its impact on the mental health and well-being of the population [41].

The literature has consistently discussed outbreaks of infectious diseases and their behavioral and psychological consequences. Most have reported mental distress [40], disruption [15], anxiety [42], cognitive impairment [43], and the stress of being infected [44] as negative psychological consequences. As far as behavioral consequences are concerned, the occurrence of infectious disease contributes to cautious health behaviors like the use of face masks [13], social distancing [45], and the pursuit of medical aid [46]. Other beneficial preventive behavioral measures include designed periods of relaxation, daily workout, diet, and self-management tools. One notable finding is that maintaining a positive psychological state helps improve the immune system [23], which could significantly decrease the likelihood of COVID-19 and its associated stress. However, a recent study in China surveyed 1257 respondents and found that most of the respondents reported mild to extreme psychological effects [44]. Greater psychological depression is also substantially correlated with current physical symptoms and poor self-rated health status [47]. Specifically, Shivakumar and Rangaraj [48] observed a clear association between mental health factors and the new WFH norm.

In fact, psychological and behavioral effects are faced by a large proportion of the workforce, more so by critical health staff [44]. According to a recent systematic analysis of the mental health effects of a catastrophe on healthcare professionals, an absence of social support and interaction and inadequate training are important risk factors for psychological morbidity [49]. Apart from those in the healthcare industry, employees in other essential business sectors have to undergo difficult employment environments during epidemics, such as the restricted provision of social and work resources and insufficient healthcare access [17,45,50].

In addition to associated risk factors, several studies have identified the factors that protect individuals against symptoms of psychological illnesses during the pandemic. A systematic review by Bentalage et al. [51] suggested that improvements to physical health, social integration, and self-efficacy can prevent mental health issues in the current situation. They further emphasized that individuals should receive support and education on new technologies. Additionally, lower levels of anxiety, stress, and depressive symptoms in the general population were shown to be correlated with the prompt distribution of revised, reliable, and relevant COVID-19 health information from the authorities [47]. Going a step further, psychiatric counseling services were given online and via voice-over-internet during the COVID-19 outbreak in China to handle mental well-being without in-person meetings [52]. Another study showed that higher confidence was correlated with better self-rated mental and physical health before the COVID-19 outbreak, suggesting that a good health status can help individuals face the unanticipated pandemic with confidence [52].

### 2.2. Holistic Technostress Model

The theoretical foundation of this study is grounded in the Holistic Technostress Model (HTM) proposed by Califf et al. [53], which sheds light on both the negative and positive aspects of technostress by categorizing technostressors into techno-distress and techo-eustress. Techno-distress occurs when an individual finds the characteristics of technology to be destructively “threatening and disturbing” [54]. On the other hand, techno-eustress assumes that individuals view technology stressors as “challenges that they are motivated to tackle because they expect that doing so is within their wherewithal and would lead to betterment” [54]. Historically, research in this domain views technostress as detrimental based on five technostress factors (e.g., techno-complexity and techno-insecurity) that are considered to lead to a variety of harmful work-related effects on individuals or organizations, such as reduced employee satisfaction and low morale in the workplace [55]. In line with the HTM, the initiation of WFH during COVID-19 posits certain challenges that are stressful for employees [12]. These stress factors hold both positive and negative outcomes [23,56]. Therefore, the HTM is a reasonable and relevant theoretical framework to assess WFH employees’ experiences and mental well-being amid the ongoing pandemic.

### 2.3. Current Research

Before COVID-19, telecommuting had been implemented in Pakistan but had not been embraced on a wide scale [57]. The emergence of COVID-19 forced a shift towards obligatory, high-intensity telework in Pakistan [58]. This hasty reform has had a significant effect on employees’ well-being across all quantitative facets of work (e.g., wages, working hours) as well as on their subjective characteristics (e.g., job satisfaction, motivation, perceived career prospects, physical and mental health). Though technology is crucial to continue our occupational, social, and educational commitments during the outbreak, evidence from the existing literature strongly suggests weighing the potential harm caused by the increased use of technology against its advantages [59]. As other studies have pointed out, limited methodological rigor, contradictory results, and inadequate effects overshadow a significant portion of the research on the effect of technology usage on physical and mental health [60]. There are thus increasing calls for researchers to understand the psychological, social, and neuro-scientific effects of the COVID-19 pandemic on mental health, especially among those who are working from home [47,61]. This is exacerbated by findings that there are broad differences in the deployment, experiences, and consequences of WFH across countries, even within the same economic field [57,58]. These findings further reinforce the need to explore the factors that affect the mental health of WFH employees during this outbreak.

## 3. Research Methods

### 3.1. Context of the Study

In February 2020, the first COVID-19 patient was confirmed in Pakistan; following that point, the country enforced the closure of office stations and employees started working from home. In Pakistan, working from home is far less common than in the West; however, the continuous rise in positive COVID-19 cases made it necessary to take drastic decisions and actions. Since 14 March 2020, both local government and companies across the nation have encouraged workers to stay at home, allowing millions of Pakistanis to experience the pros and cons of a home office for the first time. Unlike physical threats, pandemics have severe psychological impacts on the health of the general population [62]. As mentioned by WHO [10], large fractions of the population, specifically in middle- and low-income countries, are suffering from psychological, neural, and substance use disorders. Likewise, COVID-19 has impacted the mental health of the Pakistani society in one way or another. For that reason, managers must understand the factors that are contributing to the mental health issues of Pakistani WFH employees during COVID-19.

### 3.2. Study Design and Participants

The present study adopted phenomenological design of qualitative approach to explore employees’ lived experiences. The empirical phenomenological approach provided a cohesive, in-depth exploration of the experiences of employees who WFH amid COVID-19. We were also able to acquire a sense of common experience without presuming the interpretation of these experiences through the phenomenological approach [63]. Online semi-structured interviews via Zoom were conducted to collect data from the participants. Moreover, we made a concerted attempt in this study to be open-minded and impartial in understanding the shared experiences of the participants from their points of view.

### 3.3. Sampling and Data Collection

A total of 41 employees were recruited via the snowball sampling technique to participate in the study. Snowball sampling refers to a sampling technique in which initially identified participants assist the researcher in recruiting other potential participants. At the height of the COVID-19 pandemic, when it was difficult to venture out and personally identify members of the target population, the snowball sampling seemed to be appropriate in order to approach potential subjects for this study. We thus used our social networks to recruit initial study participants, who then recommended other subjects. The sample size was finalized upon reaching data saturation, wherein the emergence of new themes was no longer possible.

As shown in Table 1, the larger part of the study sample constituted male employees (65.9%). A majority of the participants were between the ages of 31 and 40 (46.3%), whereas 41.4% were younger than 30 years old and 12.1% were aged 41 and above. All the respondents were WFH employees associated with the service, IT, and manufacturing sectors. Specifically, slightly over half (51.21%) the employees were from the service sector, 36.58% were from the IT sector, and the remaining 12.19% belonged to the manufacturing sector. The employees held a broad range of roles within their business unit.

### 3.4. Procedure

Before data collection, participants were informed about the objectives and voluntary nature of the study. To maintain their confidentiality, a unique number was assigned to each participant (e.g., R1, R2, R3…) and all identifying information was removed from the transcripts. All transcripts were recorded and saved for further analysis. At the beginning of the interview, important demographic information such as the participant’s gender, age, marital status, industry, and position was obtained. A comprehensive question was then asked, i.e., “What is your overall experience of working from home?”, which was followed by a series of questions to probe the respondent’s description of their experiences. Examples of questions include “What are the key factors that make WFH challenging?” and “What do you usually do to overcome stress and stay motivated, positive, and productive?”

### 3.5. Data Analysis

To understand and interpret patterns of content and context in the data, the thematic analysis approach was adopted using a manual coding procedure [64]. Thematic analysis is a methodology that can be ideally tailored to a research project’s demands and criteria, including health and well-being research [64]. All six steps of the thematic analysis, as defined by Braun and Clarke [64], were followed in this study. First, all interviews were transcribed in an electronic spreadsheet and read repeatedly to gain familiarity with the data and identify initial ideas expressed as distinct thoughts. In the second step, we used a data-driven coding procedure by working through the text line by line to identify units of meaning and labeling them with a code that captures the identified meaning. In the next step, the analytical judgments were used to search for themes that are meaningful to the research questions. The subsequent step involved reviewing the identified themes to make necessary changes. Then, we refined the themes and sub-themes by writing a detailed analysis of each separate theme. The last step was to systematically discuss the findings.

## 4. Results

### 4.1. The Overall Experience of Working from Home

There was a great deal of variation in interviewees’ responses about their overall WFH experiences during COVID-19. The distribution of answers revealed that most respondents have a positive attitude towards WFH (51.2%), followed by respondents with mixed or strange feelings towards WFH (26.8%) and finally, those who dislike WFH (21.9%). We also noted that a higher percentage of males (36.6%) offered positive opinions about WFH than females (14.6%). In terms of marital status, positive views were specifically expressed by married males (26.9%), who appear to be more satisfied with WFH than single males. A relatively smaller proportion of married women (7.0%) were satisfied with WFH. Age-wise, a positive outlook is reflected by young employees, as the majority of young employees reported positive views of WFH. Conversely, middle-aged employees took ambivalent standpoints while older employees held negative stances in our interviews.

*“Working from home can never be completely positive for someone who has a family, especially kids, due to the distractions that an employee has to face at home from his spouse and kids” (R2).*

*“The less pressurized work environment increased my productivity, and I enjoyed this whole WFH experience” (R26).*

### 4.2. Key Factors That Make Home Office Work Challenging

Although employees value WFH, they also stumble upon significant barriers concerning their mental health. Based on our analysis, five themes (i.e., stressors) that make WFH challenging were revealed: technical issues, work-related stressors, non-work stressors, communication issues, and motivation and productivity issues.

#### 4.2.1. Technical Issues

The most prominent factor that challenges the smooth functioning of WFH is technical issues, which was highlighted by 68.5% of the respondents. Internet connectivity problems, system complexities, power cuts, resource unavailability, and system incompatibility were among the issues raised by the respondents. Those from the technology-enabled service sector referred to technical issues to a larger extent. Nonetheless, this stressor is gender agnostic as males and females were equally affected by it.

*“Sometimes the company’s web portal does not work properly. This creates a communication gap between workers and customers as well” (R6).*

*“The packages of software are really heavy…it is impossible to run them on our home computers. Also, this software is graphically intensive and cannot be managed by desktop sharing” (R10).*

#### 4.2.2. Work-Related Stressors

Increased levels of various work-related stressors were recounted by 63.4% of the study participants. In particular, respondents cited the changing dynamics of work as a major source of stress during WFH. Some shared that the absence of a professional environment, excessive work, unscheduled virtual meetings, and weekend tasks were the main work-related stressors, while others pointed to high work expectations, job insecurity, workloads, structural emptiness, and subordinate supervision as the factors responsible for mental dissonance. Reservations related to working hours were also highlighted by many respondents, irrespective of the nature of their job. In particular, older employees reported working hour issues more frequently.

*“Working from home has doubled the workload, seeing as one has to learn how to do things differently all over again” (R9).*

*“The company hasn’t given us flexible hours; we have to work from 9 am to at least 6 pm. Moreover, they haven’t compromised on the targets one bit, they say we realize the situation but still no compromise on the targets” (R24).*

#### 4.2.3. Non-Work Stressors

Apart from work-related stressors, certain non-work-related stressors were also identified by a similar proportion of the respondents (61.0%). The most cited non-work hurdle for WFH was distractions and interruption due to domestic issues or children’s presence at home (27.2%). Other stressors included time management issues, laziness due to being at home, poor work-life balance, and an inconsistent sleep schedule. Married employees perceive non-work stressors more than single employees, given that married people find more distractions at home and work best in an office setting where they cannot be bothered by anyone.

*“The major stress is family and kids, especially when kids are demanding; it is hard to make them understand that the parents are at home but not available to them” (R3).*

#### 4.2.4. Communication Issues

Some employees reported having communication issues while working from home (29.3%). No proper collaboration with superiors and peers, a lack of coordination, and non-exchange of proper feedback are the main causes behind communication gaps, which are especially prevalent for employees from the education sector. It appears that employees are highly concerned about human interactions and social relationships during the pandemic.

*“When we are working in the office, it is not only the work but the colleagues and social interaction which make the work interesting as a whole. I am a very social person. I enjoy the company of my fellow beings while working” (R19).*

#### 4.2.5. Motivation and Productivity Issues

As per our findings, the current mode of WFH has also given rise to motivation and productivity issues that weaken employees’ mental health. Over one-fifth (21.9%) of the respondents reported a significant degree of demotivation due to monotony and being bound to machines, which led to decreased productivity levels. Female employees in particular reported lacking focus due to WFH. The respondents also highlighted the importance of management support in keeping them motivated.

*“Remember, motivation comes from your higher management” (R18).*

**Figure 1:**
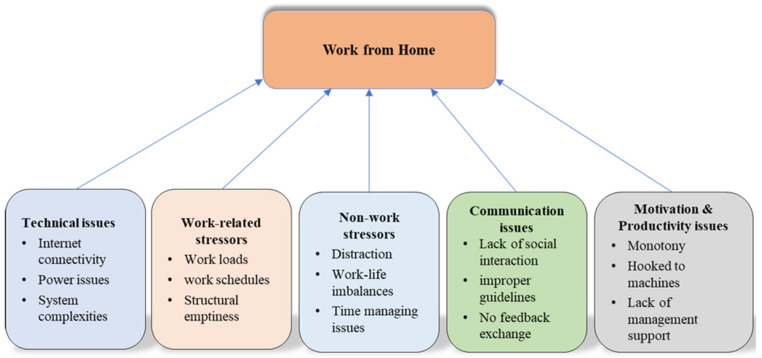
Factors affection WFH.

Figure 1 presents the five themes that emerged in this study. Based on these themes, the factors that make WFH challenging and contribute to mental health issues among WFH employees are technical issues, work-related stressors, non-work stressors, communication issues, and motivation and productivity issues.

### 4.3. Strategies to Overcome WFH Challenges

The respondents identified several stressors they face when working from home, ranging from concerns about the unavailability of resources to low motivation. To answer our second research objective, the respondents were also asked to describe the measures they take to prevent or cure the mental health problems caused by WFH during COVID-19. Based on the data analysis results, these measures were segmented into two broad categories: behavioral coping and cognitive coping.

#### 4.3.1. Behavioral Coping

To deal with mental health disturbances, participants reported behavioral coping as a means to handle stress. Behavioral coping comprises strategies intended to act on the self and the environment. Under the first sub-theme used to describe these strategies, participants appeared to place high value on cultivating healthy habits (36.6%), especially physical exercise. Other healthy habits mentioned by the employees were getting proper sleep, spending time on hobbies, adopting healthy diet plans, and taking breaks. Many also opt for leisure activities, such as watching movies, listening to songs, reading, and even blogging, while others strive to find a moment of peace by spending time on yoga, meditation, and religious activities. They also recognized the need for good nutrition and rest.

*“Activities like gardening, spending time with pets, cooking, and practicing guitar help me unwind” (R29).*

*“Going out on the lawn, having a cup of tea with some snacks, listening to music of my taste, and reading a chapter of my favorite book are the best ways for me to cope with stress” (R40).*

Under the second sub-theme of behavioral coping, participants expressed extensive experience with the use of time management strategies (40.0%), such as setting a timetable, managing tasks within the stipulated time, prioritizing work according to its significance, scheduling daily tasks, and becoming more organized (i.e., 40.0%). They further stated that simple but effective time management strategies helped them maintain mental health and optimize their work-life domains.

*“I set targets for myself and assign times for various tasks. I try to complete the tasks within time…this helps me overcome work-related stress” (R35).*

#### 4.3.2. Cognitive Coping

Strategies in which people use mental activities to respond to stress experiences are known as cognitive coping. The most frequently reported keyword pertaining to stress management among the study participants was ‘social interaction’. Social interactions, like spending time with family, virtually connecting with colleagues, and talking to friends was regarded as therapeutic to employees’ mental health. Specifically, 24% of the respondents stated that interaction with peers or colleagues was an important primary channel to relieve work-related stress. Attending mental health talks was also mentioned by participants as a way to build their awareness of mental issues.

*“Personal interactions are important; they are not just limited to serving clients well, but also to getting coworkers’ assistance and supervisory guidelines” (R41).*

Participants further commented on the value of positive emotions in dealing with mental issues. To maintain a positive attitude, employees used self-management techniques known for aiding mental health, such as deep breathing to manage anxiety. Many participants also prioritized staying calm and paying little attention to information about COVID-19 to prevent feeling upset about the unfavorable circumstances surrounding them.

*“I used to sit in my home garden for some time to feel nature and breathe in the open air and think about my tasks the next day” (R32).*

**Figure 2:**
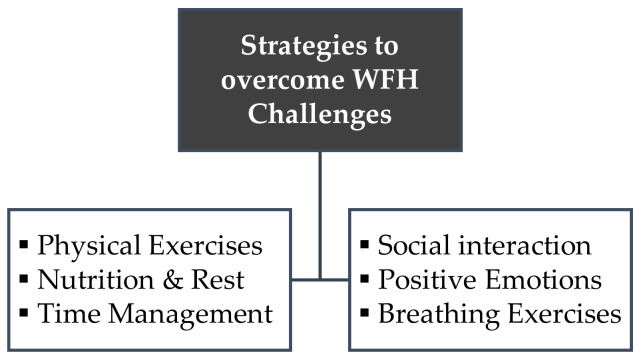
Strategies to overcome WFH challenges.

Overall, the participants of this study believed that behavioral coping and cognitive coping were two different ways to handle the mental health issues caused by working from home during COVID-19. Behavioral coping includes physical strategies applied upon oneself and the environment whereas cognitive coping encompasses the mental activities aimed to respond to stressful experiences. Figure 2 summarizes the strategies to overcome WFH challenges.

### 4.4. Recommendations for Effective and Efficient WFH

In the current scenario of the COVID-19 pandemic, both the employee and the employer are responsible for the smooth functioning of WFH with minimal stress. To this end, the study respondents offered various suggestions and recommendations on how to avoid or cope with occupational stress. These recommendations cover both employee and organizational perspectives to help policymakers and stakeholders implement effective and efficient WFH.

#### 4.4.1. Recommendations for Organizations

The respondents emphasized that organizations should take coherent measures to ensure the smooth running of business activities. The intervention of flexible arrangements was the top organization-based recommendation mentioned during the interviews (33.4%). As this is the new normal for the working world, the respondents suggested that organizations be more flexible and comprehensive in supporting their employees to maintain the latter’s well-being, such as by offering flexible working hours, reasonable work schedules, and open deadlines.

In line with flexible arrangements, effective communication was also underscored by the participants (19.0%). They argued that to maintain healthy employee relations within an organization, effective communication is mandatory as it reduces gaps and misunderstandings among individuals. Goals and targets should be well-communicated for employees to be more determined and committed to their job responsibilities. Indeed, when there is clarity in organizational communication, employees showed greater concern about their job stability, career advancement, and compensation.

It was also stressed by the participants that organizations should provide ample resources for a smooth transition to the WFH model. As this scenario is quite different from the traditional model of work, employees need equipment and technical support to create compatibility with new norms. Therefore, organizations should equip their employees with the necessary facilities and tools to keep employees focused and firmly determined towards their job responsibilities, as well as to pave the way for the smooth functioning of their duties.

Several participants further recommended organizations work harder to foster an inclusive culture where stress is not stigmatized, stress management information is accessible, and help is always available. In this regard, training could be an effective option, whereby intensive training and seminars on mental health preservation should be arranged by organizations. The establishment of a crisis team is also advocated to help employees adapt to the new working norm and address specific issues in the workforce.

#### 4.4.2. Recommendations for Employees

Considering the highly unique nature of the changes brought about by COVID-19, it is increasingly difficult for employees to remain committed to their work with full determination and passion. Accordingly, the participants of this study offered several recommendations to mitigate the factors that worsen their mental health when working from home.

First, maintaining work-life balance was highly cited by the participants. Additionally, several participants claimed to focus on setting timetables, sticking to a predefined schedule, prioritizing important tasks, taking breaks, and maintaining periodic working hours. These time management strategies evidently helped them meet deadlines and work efficiently and effectively. Participants also added that working in a well-lit space and managing a home workstation were good ways to limit distractions and focus on work.

In the epidemic situation, it is unsurprising that the interviewees exhibited concern about their physical health. Consequently, they prioritize physical exercise as a good strategy to overcome stress and keep the body fit. Different types of physical exercise like walking, playing, and jogging were mentioned by the interviewees. The employees also suggested that engaging in activities that nurture physical health had a paramount effect on their confidence, which eventually helped them combat mental health challenges.

When working remotely, loneliness, detachment, and isolation are common issues, especially for extroverted employees. Hence, networking is a key factor that can help employees heal from mental health issues during COVID-19. Networking is the development of close associations with trusted, emphatic peers and colleagues, as well as with friends and family members who are good listeners and confidence builders. Therefore, the participants recommended that interacting with friends and family members and talking to co-workers can offer mental support to alleviate stressful situations.

## 5. Discussion of the Findings

This research intended to explore participants’ perceptions of WFH, the factors that make WFH challenging for their mental health, and the strategies they employ to manage those stressors. Based on interviews with 41 WFH employees, various stressors were identified and categorized into five major themes.

Some factors have already been discussed in the literature, while others appear to be novel. Overall, the findings imply the positive attitude of employees towards WFH, especially pertaining to family and personal life. This result is in line with previous studies that have found employees’ positive sentiments towards WFH [26]. However, within our sample, more male employees reported swapping from commuting to WFH, whereas women were more likely to continue to commute to work. This is inconsistent with prior evidence that women prefer to WFH [65]. In fact, telecommuting was traditionally introduced to facilitate female employees in balancing their work and home responsibilities. The contradictory result may be because female employees’ attention span is severely strained by challenges specific to the COVID-19 pandemic, such as taking care of children who are not in school. Moreover, the results of this research indicate a generational disparity in terms of the attitude towards WFH, wherein younger employees are more inclined towards WFH than older ones. Younger employees are more responsive to emerging technologies and flexible work activities, since they are familiar with technology-rich workplaces and embrace technology as constructive. Consequently, electronic networking methods (e.g., emails and text messages) are more convenient for them to interact with. This supports the techno-eustress concept, which hypothesizes that technostress that is challenging and thrilling brings positive outcomes, including innovativeness, creativity, and productivity [53].

Apart from their positive attitude, the employees also identified several factors germane to mental health issues under WFH. The most prevalent stressor was reported to be technical issues. The literature agrees that substantial technical support is needed by employees who WFH [39]. The participants mentioned several limitations in existing technology that hamper WFH employees’ effective performance. Examples include inconsistent or non-existent cellular coverage, unplanned power cuts, and the incompatibility between old and modern technologies or between technologies introduced by different providers. Due to inadequate coverage, workers commuting from remote areas are often unable to obtain real-time company information. The technology-enabled service sector is the worst affected by technical issues, given its heavy reliance on network connectivity and complex systems. Moreover, users may require long periods to adapt to new technologies and may find that newer employees understand a technology better [54]. Users may also view the complexity of a technology as damaging to their work completion [54]. Overall, our findings echo these claims.

The second set of issues contributing to poor mental health revolves around work-related stressors. The broad range of stressors discovered illustrates the difficulty and extent of the challenging work conditions encountered by employees, such as unanticipated workloads, shifting timelines, and supplementary work. This implies that their existing workload is not an issue for WFH employees; rather, problems arise when extra tasks are sprung upon them. Work schedules are also influenced by WFH settings, as work is no longer limited to a 9-to-5 day. Employees commonly have to serve extended working hours when given the opportunity to work at home. Consistent with this, the techno-overload concept posits that using technology as a work medium forces employees to work longer and faster, leading to negative psychological responses [53]. Our findings also observed resistance from older workers regarding working hours when they began to operate remotely, possibly because they have had to shift from a predefined schedule to working throughout the day. In fact, telecommuters feel more obliged to instantly reply to work calls and work emails outside their working hours, since they have access to it from their home. This ultimately disrupts their mental health.

Non-work stressors comprise another cluster of key factors identified in this study. An important finding is that in remote working, the line between work and home gets obscured. People mostly face distractions due to home issues or the presence of children at home. During the COVID-19 lockdown, all family members have to stay home; thus, maintaining a professional environment similar to one’s office is far from ideal. Conversely, employees do not have the physical sensation of leaving the office, and hence, might never really “clock out” from their work. The blurred segregation of work and home thereby creates frustrations which ultimately affect the quality of work and induce stressed-out behaviors among employees. This finding aligns with earlier research results that WFH employees struggle with: (1) the conflict between the need for flexibility and the need for structure to preserve the work-home boundary; and (2) the benefits of segmented roles to achieve specific advantages in the telework [29,37]. The participants thus remarked that creating a balance between work and life domains is a sensitive matter, as they cannot distract themselves from the boundaries of the home while working. To handle this problem effectively, employees need to learn how to prioritize and take control of their home–work environment by awarding it the same respect as their actual office. It was also reported by the participants that being at home causes laziness, since remote workers are free to decide the amount of time they spend on tasks [66]. Some employees may not find it easy to start their work day properly; instead, the tendency for procrastination, frequent breaks, and repeated interruptions for other chores could overwhelm them [29].This supports the idea that though flexibility is beneficial to some extent, too much of it can be damaging for certain types of people [34]. Therefore, our participants emphasized developing a consistent work routine and employing time management strategies. As the home is the office for WFH employees, a line must be drawn as to which hours of the day fall under the work period and which do not [37,67].

Effective communication remains a challenge in the telecommuting context [42], [45], and has become even more demanding in the COVID-19 pandemic [67]. In this study, communication issues emerged as a key factor affecting WFH employees’ mental health. Participants reported scant collaboration with peers and superiors, inadequate coordination, and improper guidelines and feedback as major causes of stress. In the WFH environment where employees are dispersed geographically, isolation from day-to-day contact with virtual colleagues is typical. Generally, electronic communication in this regard is not as efficient as talking physically [29]. Likewise, Jaiswal and Arun [12] argued that working in virtual environments elevates feelings of professional and social isolation because it cannot fulfill the sense of belongingness, trust, team spirit, bonding, and companionship gained from physical presence and interpersonal interactions at the workplace. Participants also provided clear insights into the link between mental stress and a lack of communication, thereby confirming observations in the literature [23,45]. Interestingly, given that a lack of interaction and collaboration with peers and supervisors was the most common stressor listed by participants, correspondingly, the most common approach they mentioned to combat stress was to seek social support and encouragement from peers. This indicates that the identified stressor and coping strategy lie in the same zone. In terms of sector, communication issues were mostly faced by the education sector. Effective communication with learners is crucial in academia, making it difficult for educators to interact with students, teach technical subjects, or deal with subjects with specific spatial needs (e.g., laboratory classes) virtually.

Motivation and productivity issues were also revealed as contributors of mental stress for WFH employees. High morale is a must for one to focus on goal achievement; however, maintaining morale in remote work is more challenging than in a conventional office setting. Team spirit is also a lacking resource in telecommuting [67]. Thus, our findings are generally in line with past research results that suggest simply moving to a mobile work environment without re-evaluating the supervisor-subordinator relationship might lead to lower employee satisfaction [23]. Moreover, it was suggested by the participants that employees are self-motivated and prefer to be self-directed. As such, during the stressful situation of COVID-19, they turned to their managers for support rather than supervision. This calls for the role of supervisors to shift from supervision to facilitation [68].

Our findings are supported by the technostress theory coined by Brod [69], which is described as “a modern adaptation disease caused by an inability to interact healthily with new computer technology” [69]. Technostress is the result of the changing work dynamics and collaborative habits stemming from the use of modern information technologies in the office and at home. This theory seeks to predict and explain how the reliance on technology causes detrimental cognitive responses, such as stress, anxiety, and mental exhaustion [54,69]. Through the prism of psychosociology, Arnetz and Wiholm [70] investigated this phenomenon and discovered that technostress is a condition of mental and physiological arousal arising from the struggle to cope with technology, which is especially encountered by people who are heavily dependent on it. Complex technology, information overload, multitasking, and ubiquitous networking have been shown to lead to technostress [54,71,72]. Technostress may also be triggered by cognitive variables such as a lack of self-efficacy [73]. Tarafdar et al. [54] listed five contributors of technostress: techno-overload, techno-invasion, techno-complexity, techno-insecurity, and techno-uncertainty. Techno-overload refers to cases where people are required to work quicker and longer by using technology. On the other hand, techno-invasion represents situations in which the invasive effect of technology facilitates communication with employees anytime and anywhere. The ever-changing systems and technologies stimulate the techno-complexity, techno-insecurity, and techno-uncertainty, compelling employees to improve their skills and abilities. However, recent literature also shows that technostress has an optimistic dimension in addition to its negative ones [53].

### 5.1. Theoretical Implications

Little is known about the factors that influence the mental health of WFH employees. Taking this gap into account, the current study attempted to enhance our understanding of the key factors that contribute to WFH employees’ mental health issues amid COVID-19. This study has extended the existing literature in several ways. First, it exhibits methodological strength by employing the phenomenological approach to explore, in-depth, employees’ perceptions of the factors causing mental health issues and strategies to overcome them.

Second, though COVID-19 has exerted tremendous pressure on health systems, it is not only a physical health crisis. However, the available literature mostly emphasizes the virus’s physical repercussions, neglecting its mental health effects [19,62]. Due to the distressing COVID-19 rate, the mental health of WFH employees is very likely to deteriorate, resulting in significant mid- to long-term ramifications. The present study therefore contributes to the existing body of knowledge by offering new insights into the mental health issues of WFH employees. The results of this study provide substantial support to extant theoretical propositions.

To date, there is no holistic framework in the literature that enhances our understanding of the WFH mental health phenomenon. This is one of the few studies to explore and develop a comprehensive model of the factors that contribute to the mental health of WFH employees, which include technical issues, work-related stressors, non-work stressors, communication issues, and motivation and productivity issues. This framework can be used by future researchers to examine its potential to overcome the challenges faced by employees working from home.

Lastly, as COVID-19 rages on, countries throughout the world have been under pressure to keep their health systems well-organized and prepared to provide essential health services for everyone. In response to this call, the Pakistani government’s drive to tackle this pandemic is applauded globally [74]. Nonetheless, it should not be forgotten that WHO also highlighted mental health problems in middle-to-low income countries during the pandemic [75]. To address this issue, this study has identified the behavioral and cognitive coping strategies used by Pakistani employees to mitigate the factors that obstruct their WFH. These strategies add valuable theoretical contributions to the growing body of knowledge that seeks to prevent or cure mental health issues faced by WFH employees.

### 5.2. Practical Implications

In this study, we have identified the challenges a WFH employee generally experiences. In an applied sense, we propose a set of measures for decision-makers and policymakers who need to revisit WFH policies and practices. Such measures provide ways to improve organizational policies for the smooth functioning of WFH in a manner that safeguards the mental health of employees during the ongoing COVID-19 pandemic.

First, the findings of the study indicate the persistent presence of technical issues. To implement WFH effectively, comprehensive support is required by employees in terms of technical facilities. Employees must have the right resources and prompt access to information to work efficiently from home. As appropriate hardware devices, stable internet connections, conferencing tools, and collaboration tools are the minimum infrastructure required for WFH, organizations should invest more in enhancing these technological structures. Organizations must also adapt to new changes and bring software programs to a web-based medium so that employees can effectively WFH. This must be followed by the requisite training and technical support to permit optimum output even from home. Training and education in this regard will help employees cope with the social paradox generated by the new working arrangement and novel technologies.

Second, improper working infrastructure was found to be an influential factor affecting employees. The dearth of proper ergonomics obstructs WFH, as home offices are not easy to build and maintain. They require furnishing and equipment, which can burn a hole through one’s pocket. Organizations should support their employees in this instance by providing them with an allowance to purchase the required infrastructure (i.e., office equipment) to ensure their adaptability to the new working norm. This will eventually result in increased productivity and less stress.

Effective communication is another important factor that helps employees navigate their tasks in such crucial conditions. When employees are working from home, they are less likely to receive information from their supervisors and colleagues. Therefore, managers should arrange regular discussions with employees regarding work-related goals and keep the latter updated via the right channels. Management-level decisions should also be transparent, with employees being taken on board to avoid discrepancies. The decisions made should then be communicated at all levels to involve everyone in a positive manner. This will preserve employees’ concentration and help them stay connected to the organization’s core.

Our study’s findings emphasize the need for a proper working mechanism in this new working environment for employees to work productively from home. Organizations must provide clear guidelines to refine WFH procedures, especially regarding the “DOs and DON’Ts,” of WFH. Moreover, organizations should provide more flexibility to employees to regulate their working schedules, both in terms of working hours and submission deadlines. This will ultimately encourage an optimal balance in their work-life domains.

Another important finding of this study is that self-management alleviates stress. In this challenging situation, self-management is indeed highly required. Employees should engage in purposeful and relaxing activities to mitigate the negative effects of the current scenario. Focusing on physical health, meditation, exercise, mindfulness, and a positive attitude are some of the strategies that can be employed by workers to build confidence and prevent mental health problems.

Finally, the findings of this study reveal that WFH results in professional and social isolation, which triggers employee frustration and stress. For example, at the office, employees can go out for their lunch break, interact with each other, and return feeling ready to continue their work for the rest of the day; in contrast, with WFH, the atmosphere is the same before and after the break. Therefore, practitioners should embark on strategies to overcome employee isolation, such as by introducing unofficial virtual meet-ups where people can connect and communicate with one another. Such strategies can keep employees engaged and motivated despite being at home.

## 6. Limitations and Future Directions

Despite its strengths, the current study possesses certain limitations. First, it adopted a qualitative research design, which despite conveying a deeper understanding of participants’ experiences, limits the generalizability of the findings. Consequently, future research is advised to utilize objective analyses to inspect the occurrence and association of the psychological outcomes to reveal robust causal mechanisms.

Second, the study followed a cross-sectional design, which limits causal inferences of the results. A longitudinal design will be a good option, especially considering that the increasingly demanding situation may worsen the mental health symptoms of the workforce in the long term. It is thus worth studying the long-term psychological effects of the pandemic through longitudinal follow-ups. Similarly, this research focused only on the employee perspective. Since employees are more vulnerable to mental health issues, it may be pertinent to take into account the organizational perspective for a better understanding of the situation. Therefore, future studies should adopt an integrative approach comprising both employee and organization standpoints to develop a comprehensive picture of the prevailing scenario.

It was mentioned earlier in this paper that WFH is not as common a practice in eastern countries as it is in the West. Therefore, we believe that it is valuable to conduct a cross-cultural examination of the discovered variables. Lastly, we encourage future researchers to examine the final model of the study in different sectors and diverse work settings to assess its applicability in resolving the issues faced by WFH employees.

## 7. Conclusions

While there have been many different studies investigating the mental health issues of healthcare employees, the current study has addressed a prevalent theoretical gap by examining the mental health issues of WFH employees. In conclusion, our findings have revealed that five factors associated with WFH affect their mental health amid COVID-19. These findings imply the need for organizations to pay more attention to WFH employees’ mental health so they can better deal with the stressors stemming from the COVID-19 pandemic. By identifying the stress factors employees’ face when working from home, our results carry important practical implications for policymakers and practitioners who are interested in learning mitigation measures to safeguard the mental health of WFH employees.

## Figures and Tables

**Table 1:** Demographic information of participants (*n* = 41).

	Category	Frequency	%
Gender	Male	27	65.9
	Female	14	34.1
Age	21–30	17	41.4
	31–40	19	46.3
	41 and above	5	12.1
Marital Status	Married	25	60.9
	Single	16	39.0
Industry	Services *	21	51.21
	IT	15	36.58
	Manufacturing	5	12.19

* Service sector includeincludes education, oil and gas, digital marketing, telecommunication, and R&D institutions.

## Data Availability

The data presented in this study are available from the corresponding author upon reasonable request.
